# Coverage and Utilization in Food Fortification Programs: Critical and Neglected Areas of Evaluation^1^,^2^,^3^

**DOI:** 10.3945/jn.116.246157

**Published:** 2017-04-12

**Authors:** Lynnette M Neufeld, Shawn Baker, Greg S Garrett, Lawrence Haddad

**Affiliations:** 4 Global Alliance for Improved Nutrition, Geneva, Switzerland; and; 5 Bill & Melinda Gates Foundation, Seattle, WA

**Keywords:** coverage, utilization, large-scale food fortification, targeted fortification, salt iodization, impact, evaluation

## Abstract

The need for evidence to inform nutrition program design and implementation has long been recognized, yet the generation and use of evidence for program decision making has lagged. The results of the coverage surveys reported in this supplement highlight some of the strengths and areas for improvement of current population-based (i.e., staple foods and condiments) and targeted (e.g., foods for infants and young children) fortification programs. Among other topics, the results identify a few striking successful fortification programs whereby the majority of the food vehicle used is fortifiable and fortified, and coverage is equitable among those classified as vulnerable and not. Other programs have great potential based on very high use of a fortifiable food vehicle, including in most cases among the vulnerable, but that potential is not currently reached because of low compliance with fortification requirements. Programs were also identified whereby the food vehicle has limited potential to make public health contributions to micronutrient intake, given the low proportions of the population who consume the food vehicle in general or who consume the fortifiable food vehicle. Four key lessons were learned: *1*) the potential for impact of food fortification depends on the appropriate choice of food fortification vehicle but also on the proportion of the food vehicle consumed that is fortifiable; *2*) the design of fortification programs should be informed by the magnitude and distribution of inadequate intake and deficiency and consumption of fortifiable foods, and part of micronutrient deficiency control strategies to ensure coordination with other programs; *3*) effective quality control of fortification levels in foods urgently needs strengthening, including the many governance and other policy factors that influence the capacity, resources, and commitment to do this; *4*) periodic review of the assumptions related to dietary patterns that underpin food fortification is needed to ensure continual safe and impactful programs.

## Introduction

The need for evidence to inform program design and implementation has long been recognized for food fortification, as well as other nutrition interventions. The 2013 Lancet series and the 2016 Global Nutrition Report specifically highlight the need for sound and timely data on the nutrition problems to be addressed, and on program implementation to better coordinate and improve program quality (1, 2). In 2006, the WHO and FAO published guidelines for the fortification of food with micronutrients, the primary purpose of which was to assist countries in the design, implementation, monitoring, and evaluation of food fortification programs while taking into account appropriate evidence across each of those program stages (3). Several additional guidelines have been published that are related to specific food vehicles.

Translating that recognized need for evidence into its generation and use for program decision making, however, has lagged. To illustrate the magnitude of this gap, as of 2015, >140 countries had implemented salt iodization programs (4), 86 countries had mandated ≥1 kind of cereal grain fortification (5), and 49 had mandated the fortification of edible oils (6). Yet routine coverage data has been collected only for salt, and very few programs systematically report routine product quality data. It is precisely this paucity of information that prompted the development of the Fortification Assessment Coverage Toolkit (FACT) and the series of coverage surveys reported as part of this supplement.

The FACT is a survey instrument that was designed to assess coverage and utilization of fortified foods in both population-based (i.e., staple foods and condiments) and targeted (e.g., complementary foods for infants and young children) fortification programs, independently of any routine monitoring systems that the program may have (7). As with any survey methodology, data can be collected with any level of representativeness (e.g., national, state, urban, and rural, among others), depending on stratification, sampling design, and sample size, and can include diverse population groups. The surveys were purposefully designed to maintain costs as low as possible and to ensure rapid turnaround from data collection through analysis and reporting to provide timely and relevant information for decision making related to program improvement.

In addition to assessing coverage and utilization of fortified food vehicles, the FACT method measures actual nutrient levels in food vehicles from household or market samples to assess the adequacy of fortification in comparison with mandated levels. Furthermore, equity of coverage is assessed by identifying and classifying potentially at-risk population subgroups with the use of diverse measures of vulnerability that are associated with low micronutrient intake, poor nutrition generally, or health outcomes in low-resource settings (e.g., poverty, poor dietary diversity among women, and rural residence). An important strength of the FACT is the use of validated instruments where available to assess these components (7, 8).

From 2013 to 2015, FACT surveys were conducted to assess the coverage of large-scale fortification programs, including those for oil, wheat flour, and maize flour, in 8 countries (8). The results focused on 2 aspects of coverage, the first being the food vehicle itself, and the second being equity of coverage in the population. In relation to the food vehicle, 3 levels of coverage were assessed, i.e., whether the respondent consumed the food vehicle in the home, whether the food vehicle he or she consumed was fortifiable (i.e., industrially processed), and whether the food vehicle he or she consumed was fortified (i.e., actually contained nutrient based on analyzed samples). The quantitative assessment of nutrient content in the food vehicle also permitted comparison with mandated levels to assess the proportion of food that was adequately fortified. The assessment of equity in coverage used the 3 indicators of vulnerability mentioned above specifically poverty, poor dietary diversity among women, and rural residence.

The results for fortification of staple foods and condiments can be generalized into 3 program scenarios. The first includes a few striking successes in which the majority of the food vehicle consumed by the population studied was fortifiable and fortified, and there was equitable coverage among those classified as vulnerable and not vulnerable (e.g., maize in the 2 provinces of South Africa and oil in Abidjan, Côte d’Ivoire). The second includes programs that had great potential based on very high use of the fortifiable food vehicle, including in most cases among the vulnerable, but in which that potential was not being realized because of low compliance with fortification requirements (e.g., wheat flour in Kano, Nigeria and Senegal, and oil in Bangladesh, Rajasthan in India, Senegal, Tanzania, and Uganda). The third includes a number of programs that were identified in which the food vehicle had limited potential to make public health contributions to micronutrient intake, given the low proportion of the population who consumed the food vehicle in general (e.g., wheat and maize flour in Lagos State, Nigeria and wheat flour in Gauteng province, South Africa) or who consumed the fortifiable form of the food (e.g., wheat flour in Rajasthan, India and maize flour in Kano, Nigeria).

The FACT method was also used to assess coverage of programs that included targeted fortified products, specifically those intended for use in infants during the complementary feeding period (6–23 mo of age) (9). The programs assessed varied greatly, with 4 of 5 programs providing the complementary feeding product through some type of sales channel (e.g., regular retail shops or door-to-door sales agents), and one providing the product free of cost. Product types included fortified complementary foods and nutrient powders. The actual nutrient content of the products was not assessed as part of these surveys. The indicators assessed reflected the progression from knowledge about the existence of the product (message coverage) to use ≥1 time (contact coverage) and regular use according to a regimen shown to have a positive health effect (effective coverage) (10). Message, contact, and effective coverage were highly variable across the programs, as was the progression among them. Despite the sales model, coverage was lower among the poor in only one program (Abidjan, Côte d’Ivoire); in another program (Bangladesh), coverage was lower in those with poor infant feeding practices. The results of the surveys highlighted the fact that knowledge about the product was insufficient to ensure regular use, and the critical importance of addressing both supply and demand side constraints to achieve high coverage and use.

Finally, FACT modules were used to assess the proportion of households that used adequately iodized salt and salt containing any iodine in 10 low- and middle-income countries (11). Two key challenges for salt iodization were identified. First, whereas >50% of the population used iodized salt in all 10 countries and >80% used it in 5 countries, the proportion of the population consuming adequately iodized salt was as low as 6.2% in Niger, >50% in only 5 countries (Bangladesh, India, Indonesia, Tanzania, and Uganda), and >80% only in one country (Uganda). Second, the distribution of coverage of iodized salt was much higher in urban than in rural populations in most countries, and higher in the nonpoor than than in the poor in some countries.

In addition to providing insights into the quality of design and implementation, and ultimately the potential for impact of specific country programs, the series of studies reported here highlight a few considerations in fortification program design and implementation that merit further discussion.

## The Choice of Vehicle for Fortification Requires Information on the Consumption Pattern (or Potential Consumption Pattern) in Diverse Population Groups, and Particularly the Proportion of the Food Vehicle Consumed that Is Fortifiable

Fortification has been identified as one of the most cost-effective nutrition interventions available (12), yet the decision on what to fortify and where requires more than that simple assessment. The surveys reported by Aaron et al. (8) provide some striking examples of foods that would appear to be poor vehicles for fortification because of the very low consumption at household level (e.g., 4.3% of the population in Gauteng State, South Africa, reported consuming wheat flour). It is important to note, however, that household estimates of the staple food as such do not capture the consumption of foods purchased and consumed outside of the household that are prepared from the food vehicle (e.g., bread and other wheat flour–based products). If those products are produced with fortified wheat, as they should be if all wheat flour is mandated for fortification, actual program coverage may be substantially underestimated. Individual-level coverage and consumption of foods made from the respective food vehicles were assessed in most surveys, although the results were not included in the analysis by Aaron et al. (8).

Few low- and middle-income countries have up-to-date dietary and micronutrient deficiency data. Some tools, such as the Fortification Rapid Assessment Tool (13), exist to facilitate the collection of food data relevant to fortification programs during the design phase, yet a number of programs have been designed without that knowledge and have therefore chosen food vehicles with limited potential for impact at a population level. This results in a misallocation of resources that has serious consequences in terms of malnutrition reduction. The real potential for impact of programs can be assessed only with knowledge of the magnitude and distribution of the deficiency or inadequate intake, and then subsequent choice of vehicle for fortification that will reach those at risk.

For targeted fortification programs, additional potential barriers to acceptance of the product and the mode of use for the intended individuals within a household must be considered. As identified by Leyvraz et al. (9), there is substantial literature related to the challenges to successful targeting of foods perceived to be nutritious to single individuals within households. In this case, it is critical to understand not only the dietary gaps to be addressed, but also the potential for families to adopt a new product or dietary or child feeding pattern that will foster high coverage and use.

## Fortification Should Be Part of a Comprehensive Micronutrient Deficiency Control Strategy that Sets Clear and Achievable Goals to Assess Progress across the Lifespan of the Program and Ensures Complementarity with Other Programs If Needed

Micronutrient deficiency control strategies are needed to ensure that alternative interventions are in place to cover any segments of the population that may not be reached by food fortification. Achievable goals are also needed in recognition that a number of steps need to be in place for fortification to be successful, and that time is needed to accomplish such steps. Without such goals and some tracking of progress against them, unrealistic expectations of what can be accomplished with food fortification may prevail.

**FIGURE 1:**
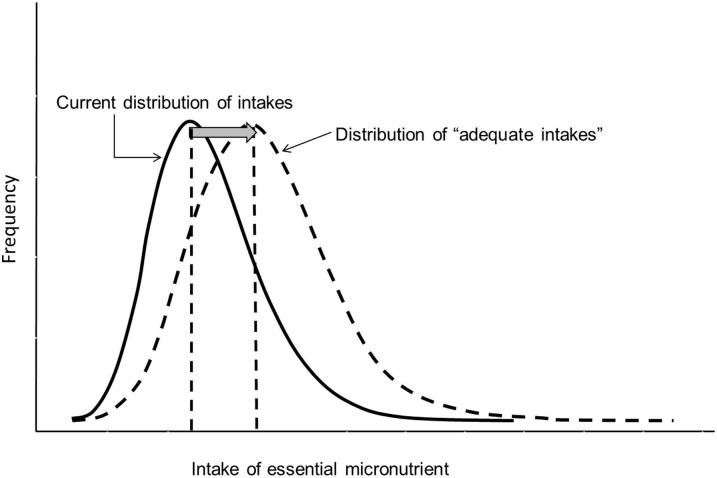
The premise of food fortification: to shift the population intake of micronutrients toward adequate values. The figure is a schematic frequency distribution of intake within a population in relation to the recommended intake for a given nutrient.

The primary premise of food fortification programs is that there is a gap in nutrient intake in a high proportion of the population and that, by fortifying foods that are commonly consumed by a large proportion of the population, the distribution of intake can be shifted toward adequate values (**Figure 1**). For nutrients such as iodine that tend to be absent from the food supply in many contexts, an appropriate goal, which in fact has been adopted by 140 countries, is the iodization of all household salt, with many countries also mandating salt iodization for the food industry, as well as for animal consumption (i.e., universal salt iodization) (5). Nonetheless, appropriate intermediate targets and a defined time frame for their achievement to track progress toward the ultimate goal and course correct where progress lags are lacking in most fortification programs, including salt iodization. Particularly notable is the inequitable distribution of coverage of adequately iodized salt, suggesting the need for redoubled efforts to reach rural and low-income populations, with appropriate targets based on the feasibility to reach them and appropriate planning for alternative sources of iodine where not feasible.

Goals should be based on realistic estimates of the time needed to establish food fortification programs. Based on the long experience with fortification, we estimate that it may take 3–5 y for government policies and legislation for fortification to be in place and for industry to acquire and implement the technology and infrastructure to fortify; this is a timeframe that should now be feasible to shorten with increased understanding a programmatic needs. This has important implications for realistic target setting and for the timeline of evaluation, particularly for programs supported by external donors, because program cycles are short.

For fortification with other nutrients that may be lacking in the diets of subsets of the population (e.g., iron) or may be impactful for the population prevention of specific outcomes even in high-income populations (e.g., folic acid fortification for neural tube defect prevention), the need for data-informed goals is essential to track program progress. Aaron et al. (8; Table 13) applied strict equity-focused criteria to judge the overall performance of the programs. Although this may be warranted on the grounds of equity, from a national public health perspective, it may not be only the poor and rural populations that have inadequate micronutrient intake and could therefore benefit from food fortification. Many high-income countries have long-standing food fortification programs based on the knowledge that the intake of essential nutrients is limited across the population (14, 15), going back to the ultimate premise of fortification shown in Figure 1. In countries with a high prevalence of low dietary nutrient intake in urban areas, fortification may be the most appropriate approach to reach them, even if that same program may not reach the rural areas. Obviously, in this scenario, alternative strategies are required for those rural areas. Only with knowledge of the magnitude and distribution of need (i.e., dietary intake and micronutrient deficiency) and the consumption of fortifiable vehicles across the population can appropriate goals be set that will permit the assessment of program progress. Included within such goals, criteria should ensure the equitable distribution of those benefits across all subgroups in the population by also clearly identifying and prioritizing for alternative interventions for those who will not be reached with fortification. This may require particular attention to salt iodization, because processed foods or condiments that contain salt are primary salt sources in many contexts, and are therefore iodized only if the government has mandated the iodization of industry salt.

## Effective Quality Control of Food Fortification Levels Urgently Needs Strengthening across All Food Vehicles and Most Countries

According to the information presented in this supplement, at this time, fortification, even when the right vehicles have been chosen (based on consumption of the fortifiable product in a high proportion of the population), is not reaching its potential for public health impact. Across all food vehicles studied (i.e., salt, oil, maize, and wheat flour) and in most countries, quality control of fortification is lacking. Concrete actions are urgently needed to understand what resource, capacity, governance, policy, or other barriers are limiting implementation, to motivate businesses to comply with fortification legislation and to ensure that governments continually monitor and enforce compliance where fortification is mandatory. Adherence to national food fortification standards could be greatly strengthened by program assessments that identify and improve procedures aligned with good practice, such as integration of fortification into existing food safety and control inspections and continual financing to support such actions, the use of an incentive and penalty schemes to ensure compliance, among others (16).

The core principles of successful food fortification are well documented (3), yet the results of these studies demonstrate that much work is needed to align program design and implementation with these. Technical assistance in low- and middle-income countries for food fortification programs should focus on identifying and overcoming issues related to capacity, governance, policy, and other barriers to successful implementation. The results reported here highlight that one of these critical gaps is the apparent lack of resources, capacity, tools, and processes to routinely and systematically monitor nutrient concentrations in fortified foods and act on that information to enforce compliance with food fortification standards.

## Data for Program Decision Making Is Not a Case of “Once and Done”

The nutrition community has had a high bar for evidence-based programing, reflected in guidance and recommendations based on randomized controlled trials. We must now embrace the reality that knowledge of what works in nutrition is necessary but insufficient to ensure that programs will be impactful. This gap can be overcome only by paying attention to detail in implementation and building evaluation into programs from their inception to identify implementation issues and inform program improvements. The surveys reported in this supplement have provided clear examples of the critical importance of coverage and utilization data to inform program design, set appropriate program goals, and track program performance and progress toward goals. To track potential for impact, data should be collected at the subnational level, including in potentially at-risk groups, with rural and poor populations included. Such activities should be considered to be core activities within programs and purposefully designed from program inception. For food fortification, clarity in expectations of what can be accomplished, and specifically the potential for distribution of benefits across diverse populations groups, is required from program design. In addition, regular quality assessment and periodic data collection during implementation to reassess the assumptions underlying program design are essential to ensure sustainable impact.

**FIGURE 2:**
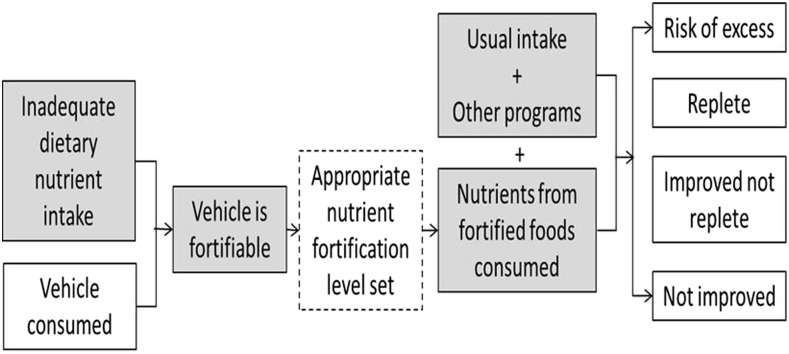
Simplified theory of change of food fortification. Gray boxes represent things that change over time and influence the relevance and appropriate targets of food fortification. The hashed box represents the point at which programs can be adjusted to adapt to changes (in addition to modifying the mix of foods that are fortified).

In addition to assuring the quality of fortification, we rely on 3 further assumptions for successful food fortification (**Figure 2**): *1*) there is a dietary gap in nutrient intake across a high proportion of the population, *2*) a high enough proportion of the population consumes a fortifiable food vehicle to warrant fortification, and *3*) for most nutrients, the fortified food is unlikely to be the only source of the nutrient, and nutrient levels in fortified foods should be set with knowledge of levels of intake from habitual foods and other sources (e.g., supplements, biofortified food vehicles, and targeted foods, among others). Over time, the burden of disease in the population changes and dietary patterns shift as a result of changes in agriculture and food industry production, place of residence, and the existence of other intervention in the population, among other factors. With these changes, the balance of impact (from no change in intake to excess intake) will change. To a great extent, these changes can be accommodated with food fortification programs by adjusting the fortification levels and the mix of different interventions to address deficiency. This has been most dramatically demonstrated with sugar fortification in Guatemala. The program began in the 1960s when the prevalence of vitamin A deficiency was severe and fortification levels were set based on sugar consumption patterns at the time (17). In recent years, there has no longer been any vitamin A deficiency in the country, but high concentrations of retinol in the liver have been detected (18). One might argue that this should be a motivation to stop the program. Yet dietary patterns show that there are still insufficient sources of vitamin A in the habitual diets (beyond fortified foods) of a large proportion of the population, and cancelling the program may put many back at risk of deficiency (19). By using the evidence of intake of other sources of vitamin A and habitual consumption of sugar, the fortification levels have now been reset to minimize safety concerns while maintaining the potential to be effective. Such tradeoffs should be assessed and managed with the use of appropriate risk-benefit approaches (20).

Dietary patterns are fast changing across Africa and Asia as populations move to more urban areas and as many foods, particularly staple foods, are being produced increasingly by consolidated large-scale industry, facilitating successful fortification and changing the vehicles that may be suitable for fortification. For example, the change in consumption of staple foods in Senegal over 10 y, including a small increase in wheat flour consumption but an increase in maize flour of >300%, is shown in **Table 1** (21). Fortification programs, as mentioned, take time to implement and should be seen as medium-term approaches to address common inadequacies in dietary intake that will require periodic adjustment to maintain relevance. Only by regular generation of coverage and utilization data can we truly understand the potential for the impact of programs. The periodicity of that data should depend on the demographic and other factors that affect the speed of change in dietary patterns in the country. Such trends can easily be tracked by commonly collected household data, such as in income and expenditure surveys (22). The FACT now provides us with a field-friendly method to track coverage and utilization among diverse population groups, which is critical to enhance evidence-informed decision making for food fortification programs.

**TABLE 1:** Mean per capita disappearance of food commodities in Senegal and 10-y trend, 1999–2009^1^

	kg/y per capita											
	1999	2000	2001	2002	2003	2004	2005	2006	2007	2008	2009	Change 1999–2009, %
Wheat	26	25.3	29.4	29.8	29.2	31.3	31.4	31.8	34.7	30.3	35.3	136
Maize	7.9	8.6	9	11.6	12.9	23.7	32.1	27	26.6	29.1	29	367
Rice	72.7	76	67	73.7	75.3	73.1	72.2	69.6	76.4	75.9	71.5	98

1 Data from FAO food balance sheets (21).
